# Microstructure and Doping/Temperature-Dependent Photoluminescence of ZnO Nanospears Array Prepared by Hydrothermal Method

**DOI:** 10.1186/s11671-018-2622-2

**Published:** 2018-07-25

**Authors:** Shiwei Shi, Peihong Wang, Jingbiao Cui, Zhaoqi Sun

**Affiliations:** 1 School of Physics & Material Science, Anhui University, Hefei, 230601 China; 2 Co-operative Innovation Research Center for Weak Signal-Detecting Materials and Devices Integration, Anhui University, Hefei, 230601 China; 3 Department of Physics and Material Science, University of Memphis, Memphis, 38152 USA

**Keywords:** ZnO, Al doping, Photoluminescence, Doping/temperature dependence

## Abstract

Al-doped ZnO nanospears were prepared by a hydrothermal method. The crystalline structure and photoluminescence properties of ZnO nanospears were characterized for investigating the effect of Al doping on the properties of ZnO nanospears. ZnO nanospears grow preferentially along the *c*-axis and have a fine tip. Al doping reduces the length of ZnO nanospears. In room temperature, photoluminescence spectra of Al-doped ZnO nanospears, a near band edge emission (~3.16 eV), and a violet emission (~2.91 eV) exhibit a strong doping-dependent characteristic and a temperature-independent characteristic, while deep level emission peak shows a temperature-dependent characteristic. In variable temperature, photoluminescence spectra near band edge emission (~3.31 eV) and its fine structures were observed when the measurement temperature is less than 57 K, and it shows an obvious temperature-dependent characteristic. The thermal quenching of this near band edge emission should be attributed to exciton scattering by defects and the presence of a high concentration of defects in Al-doped ZnO nanospears.

## Background

Recently, one-dimensional semiconductor nanostructures have been one of the focuses of current research in physics, chemistry, and materials science due to their significance in both fundamental knowledge and technological applications [1]. Among those semiconductor nanomaterials, ZnO has received broad attention for potential applications in short wavelength optoelectronic devices, owing to its wide direct band gap of 3.37 eV at room temperature and large exciton binding energy of 60 meV [2]. More attractively, nanostructured ZnO has a diverse group of growth morphologies, which can be widely used to construct nanoscale devices for various needs. In order to develop devices with desired functionality, the properties of ZnO have been tuned by various approaches [3]. Among them, doping is an effective way to change or adjust the electronic and optical properties of materials [2]. For optoelectronic applications, doping of ZnO has to be done to achieve the ideal properties and device’ performance [4, 5]. It has been proved by many reports that the substitution of Zn^2+^ ions with group III ions (B^3+^, Al^3+^, Ga^3+^, and In^3+^) [6–8] generates extra electrons to obtain n-type ZnO. Among these dopants, Al is the commonly used dopant due to its small ionic radius and low cost. Al doping in ZnO lattice improves the donors’ concentration and introduces new energy levels in the bandgap of ZnO with enriched properties such as better conductivity, high transparency, extremely stable field emission property, etc. [4].

ZnO nanostructures can be synthesized via vapor deposition or the hydrothermal method. With the hydrothermal method, it is possible to form well-aligned doped ZnO nanostructures and to control their size and morphology by varying the reaction species and synthetic conditions [1]. In addition, this method allows the ZnO nanostructures to be prepared at low temperature with simple equipment, making the process more effective and convenient. There have been many reports of Al-doped ZnO (AZO) film or nanostructures synthesized by the hydrothermal method [9–13]. But most of these reports are mainly concerned about the morphology control [9, 10], the electrical properties [5], and the applications in gas sensor [11], pH-sensor [12], or dye-sensitized solar cells [13] of AZO nanostructures. There are few reports concerned about the effects of Al doping on the photoluminescence (PL) spectrum, especially the temperature-dependent PL characteristics, of AZO nanostructures prepared by hydrothermal method.

In this study, aluminum nitrate and zinc nitrate were used to prepare AZO nanostructures by hydrothermal method. By adjusting the pH value of precursor solution to 10.0, the AZO nanospears (nanorods with fine tips) were prepared. Al doping has negative effects on the average length of AZO nanospears. In the PL measurement results, two emissions, a near band edge emission (~3.16 eV) and a violet emission (~2.91 eV), of AZO nanospears show a strong doping-dependent characteristic and a temperature-independent characteristic, while other emissions shows a opposite characteristic. Excitonic emission (~3.31 eV) and its fine structures was observed when the measurement temperature drops to 10 K, and it shows an obvious temperature-dependent characteristic. These results were discussed in detail.

## Methods

### Preparation of Samples

The AZO nanospears were prepared by the hydrothermal method on the glass substrate with a ZnO seed layer. ZnO seed was prepared by a sol-gel method which was described subsequently. Then, 8.76 g zinc acetate dehydrate (Zn(CH_3_COO)_2_ 2H_2_O) was dissolved into 80 mL ethylene glycol monomethyl ether at room temperature. Mono-ethanol amine was used as the stabilizing agent. The molar ratio of mono-ethanol amine to zinc acetate dehydrate was kept as 1.0. They were mixed rapidly, and stirred at 60 °C for 120 min, then cooled to room temperature. The solution served as the coating sol after being kept for 1 day. The sol was then spin-coated on the substrate at 1500 rpm for 18 s, and 3000 rpm for 30 s. After spin coating, the substrates were heated at 150 °C for 10 min to remove the solvent and this procedure was repeated two times. These as-coated films were annealed at 500 °C for 2 h in air and then cooled down to room temperature. The seeded glass substrates are vertically positioned in a 50 mL Teflon-lined stainless steel autoclave which contains 40 mL aqueous solutions of zinc nitrate (Zn(NO_3_)_2_, 20.0 mmol), aluminum nitrate (Al(NO_3_)_3,_ 0–4.8 mmol), hexamethylenetetramine ((CH_2_)_6_N_4_, 10.0 mmol), and aqueous solution of ammonia (NH_3_·H_2_O, 0.5 mL). So the Al(NO_3_)_3_ concentration in the precursor solutions are from 0 to 0.12 M (M = mol/L). The autoclave is airtight and put into a constant temperature drying oven. The ZnO nanospears were formed at 368 K for 1 h. After growth, the substrates are taken out of the solution and rinsed several times with deionized water, and then dried in air at 333 K. For convenience, AZO nanospears prepared with 0.0, 0.02, …, 0.12 M Al(NO_3_)_3_ will be called 0 M ZnO, 0.02 M AZO, …, 0.12 M AZO, respectively. The mechanism for the growing of the ZnO and AZO nanospears can be summarized in the following equations [10, 14]:

1 $$ {\left({\mathrm{CH}}_2\right)}_6{\mathrm{N}}_4+6{\mathrm{H}}_2\mathrm{O}\to 6\mathrm{HCOH}+4{\mathrm{N}\mathrm{H}}_3 $$

2 $$ {\mathrm{NH}}_3+{\mathrm{H}}_2\mathrm{O}\to {{\mathrm{NH}}_4}^{+}+{\mathrm{OH}}^{\hbox{-} } $$

3 $$ \mathrm{Zn}{\left({\mathrm{NO}}_3\right)}_2\cdot 6{\mathrm{H}}_2\mathrm{O}\to {\mathrm{Zn}}^{2+}+2{{\mathrm{NO}}_3}^{-}+6{\mathrm{H}}_2\mathrm{O} $$

4 $$ \mathrm{Al}{\left({\mathrm{NO}}_3\right)}_3\cdot 9{\mathrm{H}}_2\mathrm{O}\to {\mathrm{Al}}^{3+}+3{{\mathrm{NO}}_3}^{-}+9{\mathrm{H}}_2\mathrm{O} $$

5 $$ {\mathrm{Zn}}^{2+}+4{\mathrm{OH}}^{-}\to \mathrm{Zn}{\left(\mathrm{OH}\right)}_4^{2-}\to \mathrm{Zn}\mathrm{O}+{\mathrm{H}}_2\mathrm{O}+2{\mathrm{OH}}^{-} $$

Zn^2+^ are known to react readily with OH^−^ to form more soluble Zn(OH)_2_ complexes, which act as the growth unit of ZnO nanostructures [3, 10, 14]. Finally, ZnO nanospears is obtained by decomposition of Zn(OH)_4_^2−^. Therefore, the key parameter for the growth of ZnO nanospears is controlling the supersaturation of the reactants as Eq. (5). Also, (CH_2_)_6_N_4_ plays a very complicated role in the solution during the hydrothermal method, and it supplies OH^−^ to the Zn^2+^and Al^3+^ to form Zn-O and Al-O bonds here, respectively [15]. Thereby, Al doping of the ZnO lattice was achieved by interstitial and/or substitution reaction. As the pH value of precursor solution is an important factor on morphological control of ZnO nanostructures [9, 15], it was improved to about 10 by adding 0.5 mL NH_3_·H_2_O in order to get ZnO nanospears.

### Characterization

The crystal structure and morphology of the AZO nanospears were investigated by X-ray diffraction (XRD, MXP18AHF, Mark, Japan) and field emission scanning electron microscopy (SEM, S-4800, Hitachi, Japan). The average length of nanospears was measured by surface profiler meter (XP-1, Ambios, USA) using line scan model from the surface of nanostructures to substrate. The compositions were measured by X-ray photoelectron spectroscopy (XPS, ESCALAB 250, Thermo-VG Scientific, USA). The PL measurements were performed on a spectrograph (Horiba Jobin Yvon iHR320, France) using a He-Cd Laser (Kimmon 1K Series He-Cd Laser, Japan) as the excitation light source. The excitation wavelength was 325 nm. The variable temperature PL was measured by cooling the samples down to the desired temperatures in a cryostat. The measurement temperature was varied from 10 to 297 K.

## Results and Discussion

### Microstructure and Morphology

**Fig. 1:**
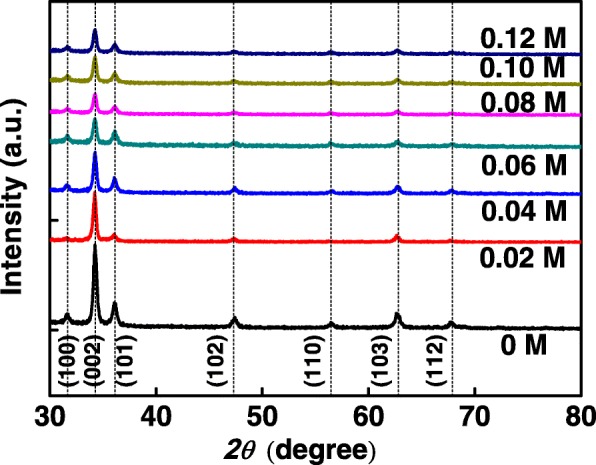
The XRD patterns of AZO nanospears

**Fig. 2:**
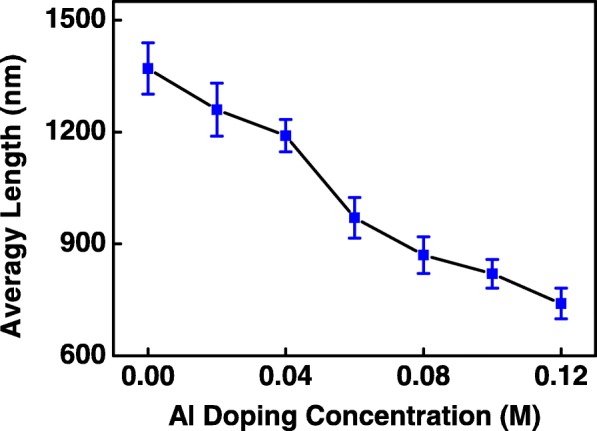
The average length of AZO nanospears

The XRD diffractograms of AZO nanospears are shown in Fig. 1. All samples have a hexagonal wurtzite structure with a preferential growth along the (002) orientation [5]. The growth of the AZO nanospears is influenced by Al(NO_3_)_3_ in the precursor solution. The higher the Al(NO_3_)_3_ concentration is, the weaker the intensity of the XRD patterns is. Such influence on ZnO growth may be attributed to the decrease of pH value in the solution due to the addition of Al(NO_3_)_3_. As was reported, the alkalinity of the precursor solution is beneficial for the growth of ZnO nanostructures [10]. The pH value of the precursor solution without Al(NO_3_)_3_ is 10.16, and that of the precursor solution with 0.10 M Al(NO_3_)_3_ decreases to 9.60. The decrease of the pH value is disadvantageous to the growth of Al-doped ZnO nanospears and weakens the intensity of the XRD peaks of AZO nanospears. Similar result was reported in [2]. The average length of 0 M ZnO, 0.02 M AZO, …, 0.12 M AZO nanospears were 1370, 1263, 1190, 972, 870, 819, and 740 nm, respectively, as shown in Fig. 2. It is shown that the average length of AZO nanospears were decreased with increasing Al(NO_3_)_3_ concentration. This result is consistent with that of the XRD diffractograms.

**Fig. 3:**
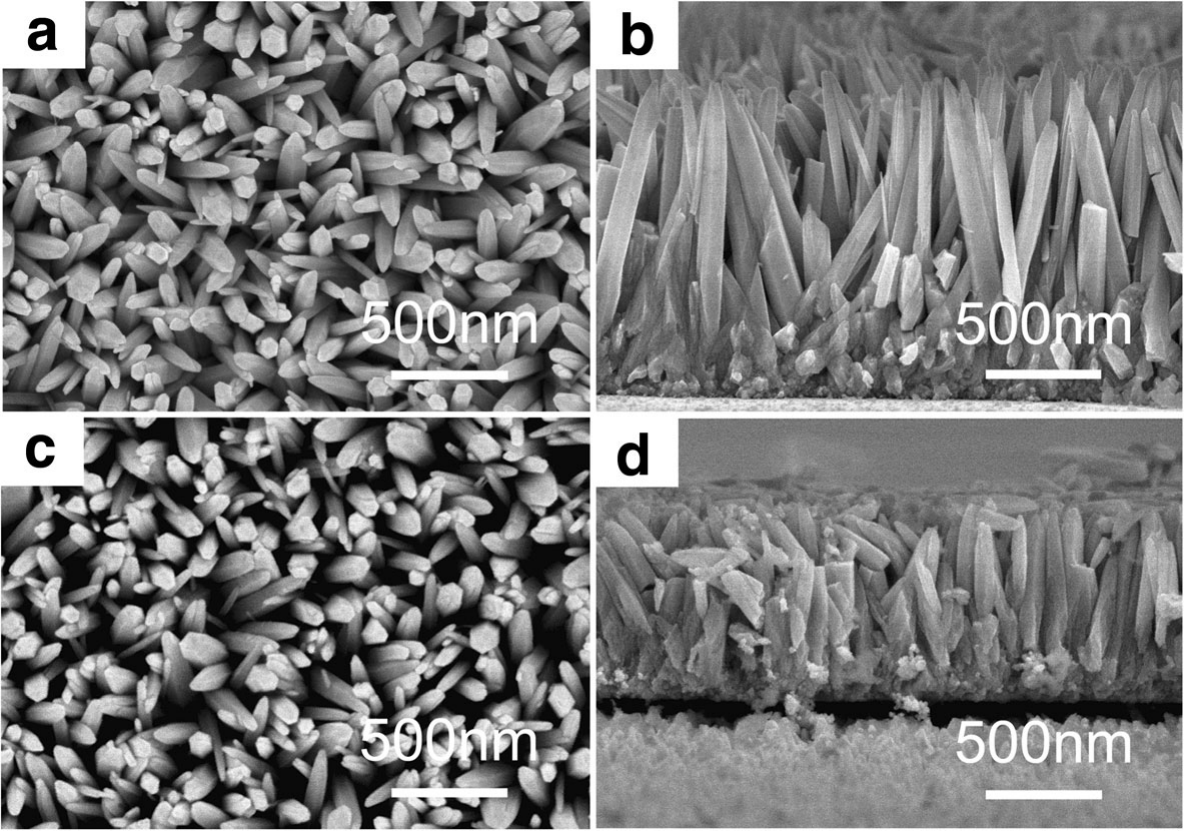
The SEM images of 0 M ZnO and 0.08 M AZO nanospears, **a** top view and **b** side view of 0 M ZnO nanospears, **c** top view and **d** side view of 0.08 M AZO nanospears

The SEM images of 0 M ZnO and 0.08 M AZO nanospears are shown in Fig. 3. It is shown that AZO nanospears have a regular appearance as a hexagonal spears with a fine tip. Most of the AZO nanospears have a diameter of about 100 nm. The average length of AZO nanospears prepared without Al(NO_3_)_3_ is about two times higher than that of 0.08 M AZO nanospears. ZnO nanostructures have been presented for the good conductance and high crystal quality, which could be expected to have lower turn-on field and higher emission current [16]. Such characteristics of nanostructures (nanorods, nonawires, nanosheets, etc.) have been reported in many reports [17–19]. Yang has reported the good field-emission characteristics of undoped ZnO and AZO nanostructures hydrothermally synthesized at low temperature [16]. As the similar microstructure, ZnO nanospears in our reports could be considered as a potential field emission materials.

### Compositions

**Fig. 4:**
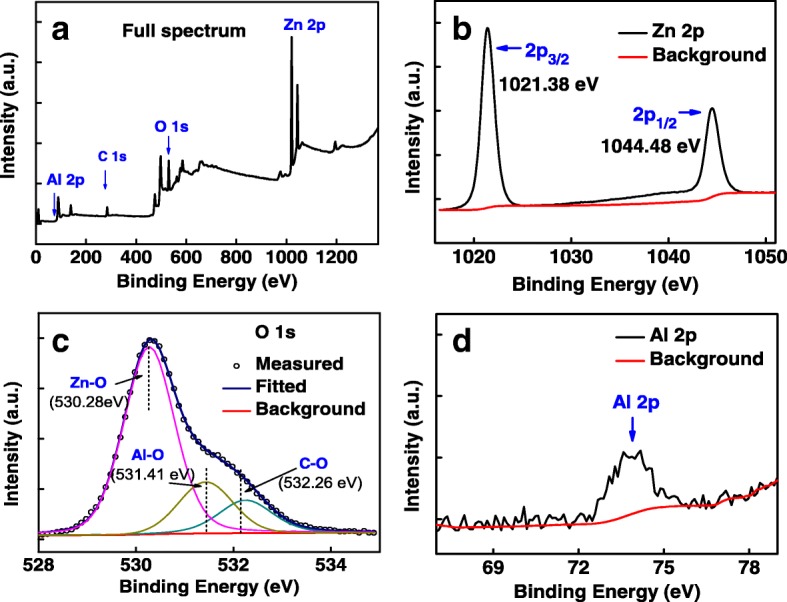
The XPS spectra of 0.12 M AZO nanospears, **a** full spectrum, **b** Zn 2p, **c** O 1s, and **d** Al 2p

The compositions of AZO nanospears were characterized by XPS. Figure 4 shows the XPS spectra of 0.12 M AZO nanospears. The overall survey shows the typical peaks from Zn, O, and C. The fine scan of Zn 2p, O 1s, and Al 2p are also measured and shown in Fig. 4b–d. The two peaks located at 1021.38 and 1044.48 eV belong to Zn 2p_1/2_ and 2p_3/2_ [20]. The O 1s peak can be deconvoluted into three peaks at 530.28, 531.41, and 532.26 eV which can be assigned to O bonded to Zn, Al, and C, respectively [16, 21]. The Al 2p peak at 73.9 eV is weak but clearly present in the XPS spectra, which can be attributed to the Al-O bonds [20]. It shows that Al was doped into ZnO matrix by this hydrothermal method. The composition of AZO nanospears can be estimated by the XPS data measured from the surface of AZO nanospears, listed in Table 1. It is shown that the ratio of Al atoms to others in AZO nanospears prepared with 0.12 M Al(NO_3_)_3_ is 1.29%. The Zn/O atomic ratio is about 34.25/34.66.

**Table 1:** The calculated composition of 0.12 M AZO nanospears

Name	Al	C	O	Zn
Atomic%	1.29	29.8	34.25	34.66

### Photoluminescence Properties

**Fig. 5:**
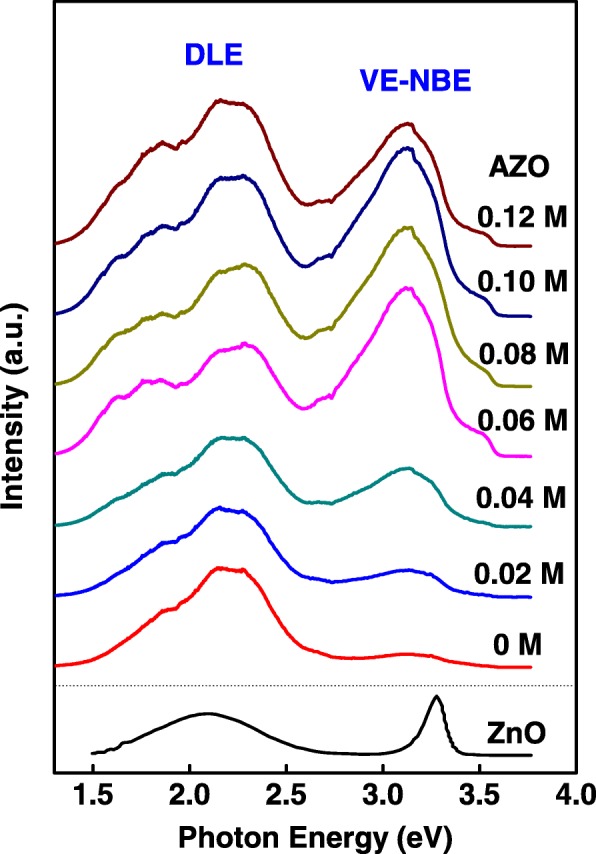
Room temperature PL spectra of AZO nanospears. The spectra are vertically offset for clarity. The spectrum in the bottom of the figure is the PL spectrum of undoped ZnO rods in [2]

**Fig. 6:**
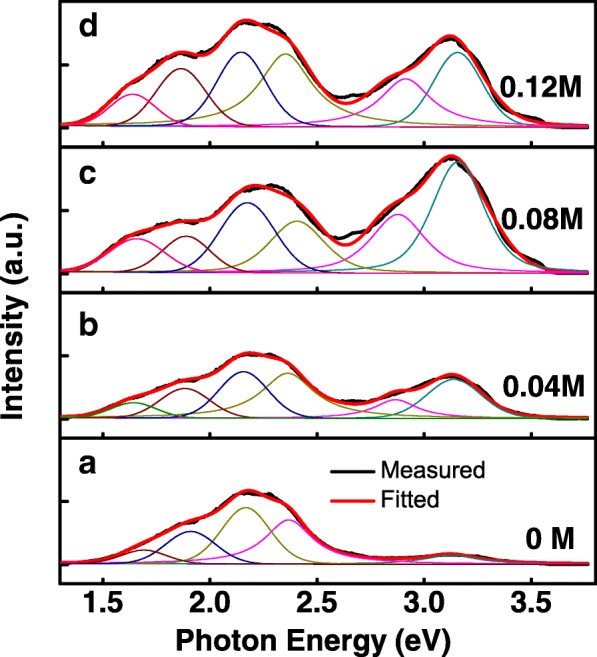
Peak fitting of PL spectra of four samples, **a** 0 M ZnO, **b** 0.04 M AZO, **c** 0.08 M AZO, and **d** 0.12 M AZO nanospears

Room temperature PL spectra of the AZO nanospears are shown in Fig. 5. All the spectra were smoothed for removing noise. The spectra are vertically offset for clarity. PL spectrum of undoped ZnO rods in [2] was plotted for comparison. In PL spectra of AZO nanospears, two broad emission peaks were observed. One is an overlap peak of near band edge (NBE) emission peak and violet emission (VE) peak, the other is a broad deep level emission (DLE) peak in the visible region. The DLE emission peak is similar to that in [2]. But the NBE emission at 3.28 eV in [2], which was usually attributed to exciton emission, is not observed in PL spectra of AZO nanospears. It will be discussed later. Al doping has different effect on VE-NBE emission peak and DLE emission peak. An obvious doping-dependent characteristic was observed on VE-NBE peak while such characteristic was not found on DLE peak. In order to discuss that in detail, peak fitting of PL spectra of samples (0, 0.04, 0.08, and 0.12 M) were carried out and shown in Fig. 6. It is shown that in PL spectra of 0.04, 0.08, and 0.12 M sample, VE-NBE peak can be deconvoluted into a VE peak (~2.91 eV) and a NBE emission peak (~3.16 eV). However, in PL spectrum of 0 M ZnO nanospears, there is only a NBE emission peak (~3.16 eV) but no VE peak. While Al doping concentration increases from 0 to 0.12 M, VE emission arises and the intensity of VE and NBE emission is synchronously enhanced. By comparing with the photoluminescence spectrum of bulk ZnO [22], it is found that the NBE emission (~3.16 eV) should be attributed to the donor-acceptor-pair transition or its phonon replica [22]. Al doping leads to the increase of donor-acceptor-pair concentration so that the NBE emission (~3.16 eV) is strongly enhanced by Al doping. VE emission is also observed by Gang [9] and Yang [16]. Both of these reports supposed the VE emission were attributed to the radiative transition between defects’ energy level and the valence band. It was reported that Al doping atoms in ZnO is a shallow donor [23]. As VE emission were emerged and strongly enhanced by Al doping, it was supposed that the “defects” should be the Al doping atoms in ZnO. Moreover, the VE emission should be attributed to the radiative transition from Al doping atoms energy level to the valence band. The DLE emission peak was fitted into four components (two red emissions at 1.69 and 1.90 eV, two green emissions at 2.16 and 2.36 eV). These DLE emissions were usually observed in PL spectra of ZnO nanostructures or films and were discussed frequently [16, 22, 24]. These DLE emissions were usually attributed to the deep level intrinsic defects of ZnO (i.e., oxygen vacancies, oxygen interstitials, and zinc vacancies) [16, 22, 24]. In our report, Al doping has no obvious effects on this DLE emission peak. In addition, there is a clear boundary at 2.6 eV for VE-NBE emission peak and DLE emission peak. In the two side of the boundary, the changes of the two peaks with the different Al concentration are very different. It shows that the origin of the two peaks is different. As mentioned above, we suppose that the VE emission of AZO nanospears should be mainly attributed to Al doping atoms in ZnO and the DLE emission be attributed to the intrinsic defects.

**Fig. 7:**
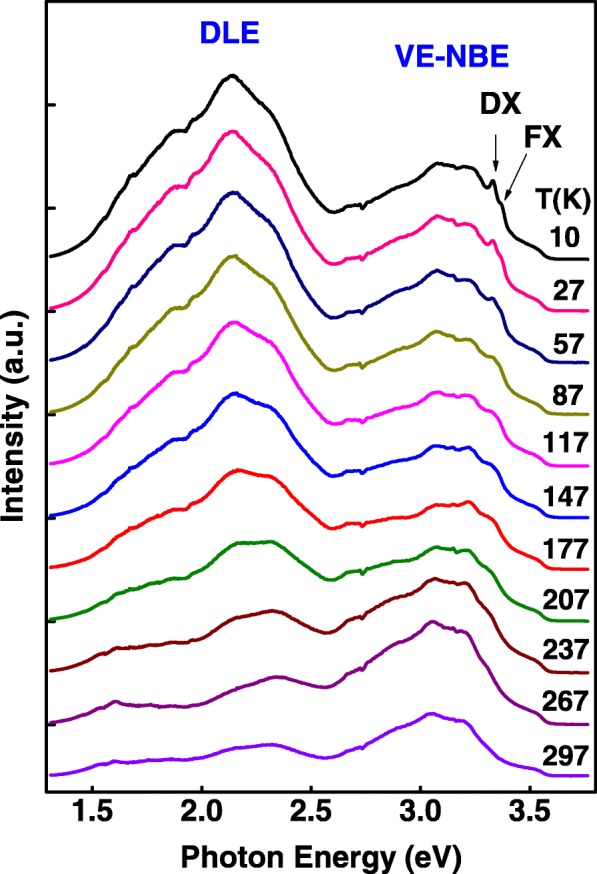
Temperature-dependent PL spectra of 0.08 M AZO nanospears

**Fig. 8:**
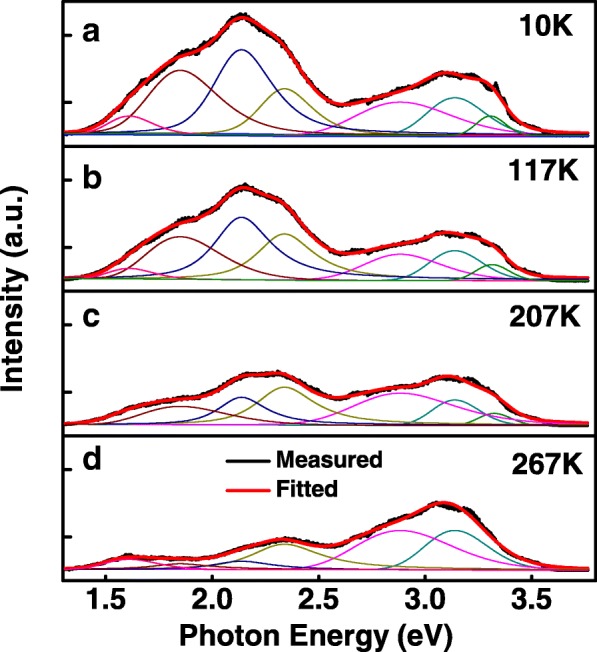
Peak fitting of PL spectra of 0.08M AZO nanospears measured at **a** 10, **b** 117, **c** 207 and **d** 267K

The variable temperature PL spectra of ZnO nanospears prepared with 0.08 M Al(NO_3_)_3_ are shown in Fig. 7. In order to discuss these PL spectra in detail, peak fitting of PL spectra measured at 10, 117, 207, and 267 K were carried out and shown in Fig. 8. In Fig. 7, there is a clear boundary at 2.6 eV between VE-NBE peak and DLE peak. In the two side of the boundary, the changes of these two peaks are different. A strong temperature dependence of DLE peak was observed. The intensity of DLE peak decreased quickly when the temperature increased from 10 to 297 K, but the intensity of NBE emission peak changed a little. The quenching of the DLE peak should be attributed to the quick increase of the non-radiative transition probability as temperature increasing [25]. As measurement temperature increases from 10 to 297 K, the NBE emissions slightly shift to lower energies, which is suggested to be caused by the thermal expansion of the lattice and changing electron-phonon interactions, and therefore the decrease of the band gap [26]. In Fig. 8, VE-NBE emission peak measured at 10 K was fitted into three components (a VE emission at 2.91 eV and two NBE emissions at 3.16 and 3.31 eV). As the measurement temperature increases, the VE emission at 2.91 eV and NBE emissions at 3.16 eV exhibit a temperature-independent characteristic. Similar result was observed in Cui’s reports [27]. This could be caused by the defect scattering effect in ZnO nanospears, which completely smears the thermal quench process in the PL spectra [27]. Fine structure was observed in NBE emission when measurement temperature is below 57 K. Similar fine structures were observed at low temperature in other reports [28, 29]. The NBE emissions around 3.31 eV are usually attributed to donor-bound excitons (DX), free excitons (FX), or the two-electron satellite [22, 30]. In the fine structures, one emission at 3.33 eV and the other weak emission at 3.37 eV were observed. These two emissions were usually assigned to DX and FX respectively [27]. As the measurement temperature increasing from 10 to 297 k, the fine structures disappeared and NBE emission at 3.31 eV became weak until it quenched when measurement temperature exceed 267 K. This result should be attributed to exciton scattering by defects, and the presence of a high concentration of defects results in a thermal quenching effect in the NBE emission [27].

## Conclusions

AZO nanospears were prepared by a hydrothermal method. AZO nanospears grow preferentially along the *c*-axis and have a fine tip. Al doping reduces the length of AZO nanospears. In the PL spectra of AZO nanospears, a NBE emission (~3.16 eV) and a VE emission (~2.91 eV) show a strong doping-dependent characteristic and a temperature-independent characteristic which could be caused by the defect scattering effect in ZnO nanospears. DLE emission peak shows a temperature-dependent characteristic which should be attributed to the quick increase of the non-radiative transition probability as temperature increasing. In variable temperature PL spectra, excitonic emission (~3.31 eV) and its fine structures was observed when the measurement temperature drops, and it shows an obvious temperature-dependent characteristic. The NBE emission (~3.31 eV) quenched if the measurement temperature exceed 267 K. The thermal quenching of this NBE emission should be attributed to exciton scattering by defects and the presence of a high concentration of defects results in a thermal quenching effect in this NBE emission.

## Abbreviations

AZO: Al-doped ZnO
DLE: Deep level emission
DX: Donor-bound excitons
FX: Free excitons
NBE: Near band edge
PL: Photoluminescence
SEM: Scanning electron microscopy
VE: Violet emission
XPS: X-ray photoelectron spectroscopy
XRD: X-ray diffraction
